# Supplementary material for: Basin-wide sea level coherency in the tropical Indian Ocean driven by Madden–Julian Oscillation
Source: Nat Commun. 2019 Mar 19;10:1257. doi: 10.1038/s41467-019-09243-5 (PMC6425029; doi:10.1038/s41467-019-09243-5)
Supplement: Supplementary file 1 — Supplementary Information [file 41467_2019_9243_MOESM1_ESM.pdf]

Supplementary Information

for

"<u>Basin-wide sea level coherency in tropical Indian Ocean driven by Madden-Julian Oscillation</u>"

Rohith et. al.

Supplementary Figures:

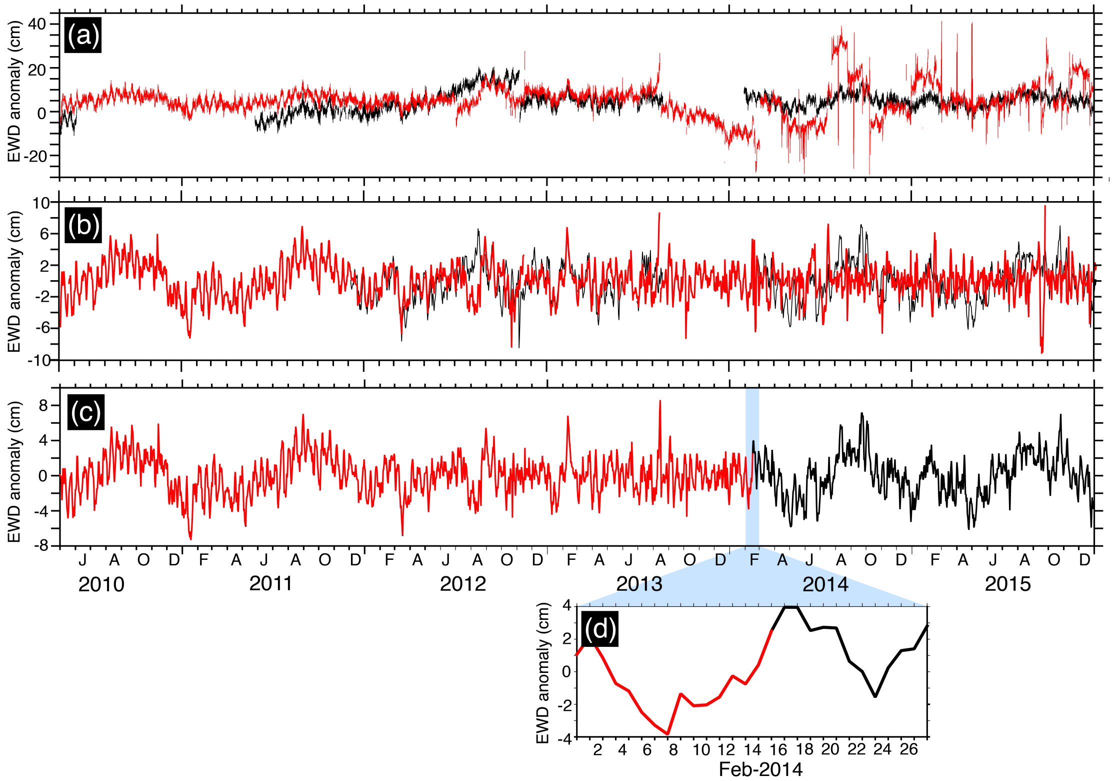

Supplementary Figure 1 | Data processing stages of EWD at BP-BoB. (**a**) Hourly de-tided EWD anomaly at BP-BoB1 (red) and BP-BoB2 (black). (**b**) De-tided EWD anomaly after removing spikes and trends at BP-BoB1 (red) and BP-BoB2 (black). (**c**) Combination of the two BPRs – BP-BoB1 (red) and BP-BoB2 (black) - to create a continuous time series of EWD anomaly which was eventually used in our analysis. J, A, O, D, F, A in the x-axis labels denote June, August, October, December, February and April respectively in panels (a)-(c). (**d**) EWD anomaly for Feb, 2014 at BP-BoB1 (red) and BP-BoB2 (black) where the concatenation between the two time series was done.

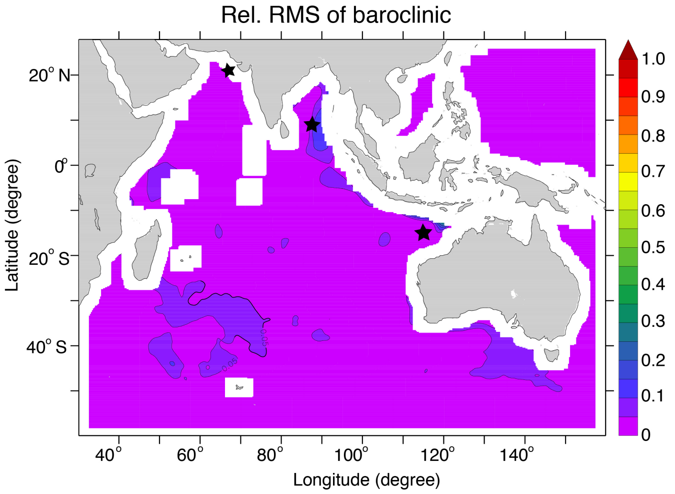

Supplementary Figure 2 | Oceanic bottom pressure versus barotropic bottom pressure. Standard difference of the model barotropic bottom pressure and total bottom pressure, normalized by the standard deviation of total bottom pressure. It is computed from the UV experiment, 5°×5° spatially smoothed and intraseasonally filtered. The location of the three BPRs are marked by a black star symbol.

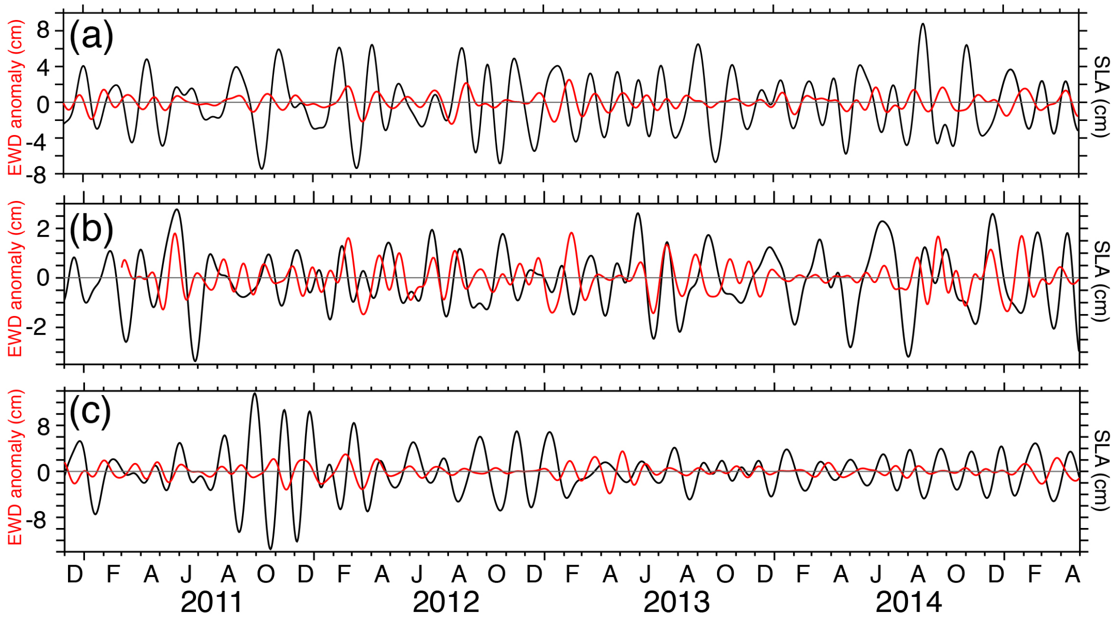

Supplementary Figure 3 | Intraseasonal EWD anomaly versus intraseasonal SLA from AVISO. Time series of intraseasonal EWD anomaly (red) and intraseasonal SLA from AVISO (black) at (a) BP-BoB (b) BP-AS and (c) BP-NWAB during the period December 2010 – April 2015. J, A, O, D, F, A in the x-axis labels denote June, August, October, December, February and April respectively in panels (a)-(c). y=0 line is plotted in (a), (b) and (c) for clarity.

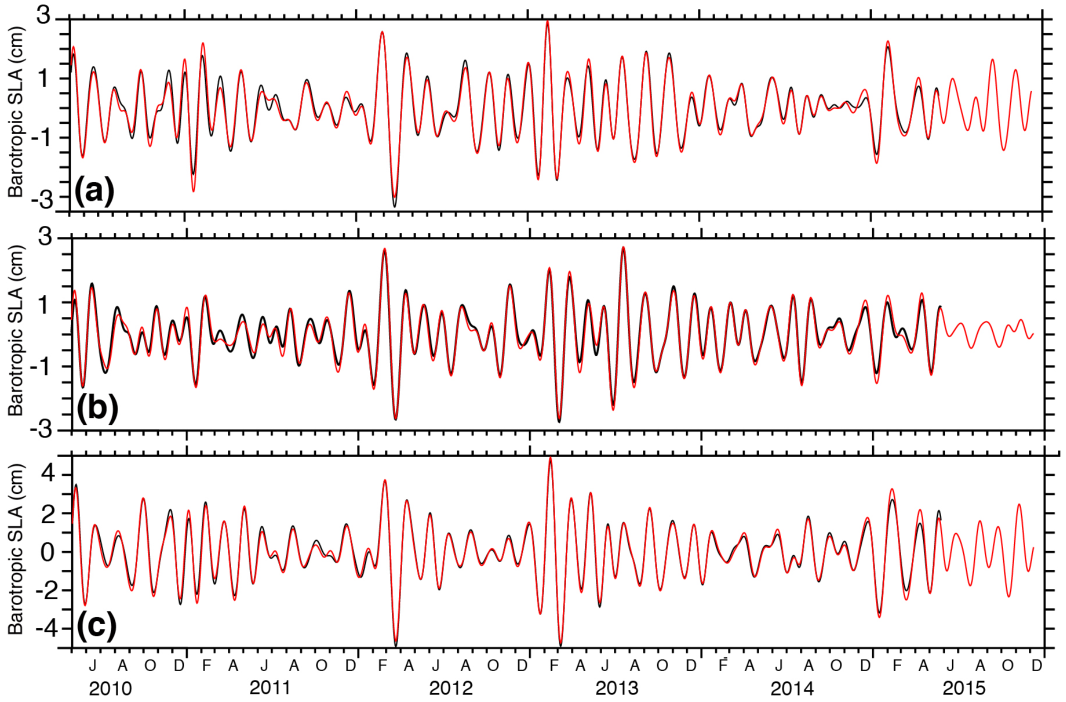

Supplementary Figure 4 | Effect of atmospheric pressure on barotropic SLA. Barotropic SLA obtained from MOM4p1 with real time-evolving atmospheric pressure (UVP experiment, black) and uniform time-independent pressure of 1025 mb (UV experiment, red) at (**a**) BP-BoB, (**b**) BP-AS and (**c**) BP-NWAB. J, A, O, D, F, A in the x-axis labels denote June, August, October, December, February and April respectively in panels (a)-(c).

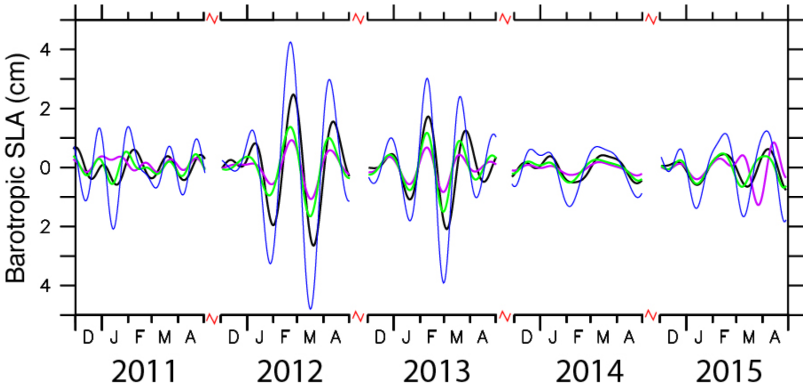

Supplementary Figure 5 | NWAB-FB sensitivity experiment. Time series of Intraseasonal barotropic SLA obtained from NWAB-FB experiment with varying depth conditions (pink - 5300 m, green-3000 m, blue-1100 m) were compared with NWAB experiment (black) at BP-BoB during the period December 2010 – April 2015. y=0 line is plotted for clarity. Supplementary Table 1 contains the details of the experiments. D, J, F, M, A in the x-axis labels represents December, January, February, March and April respectively.

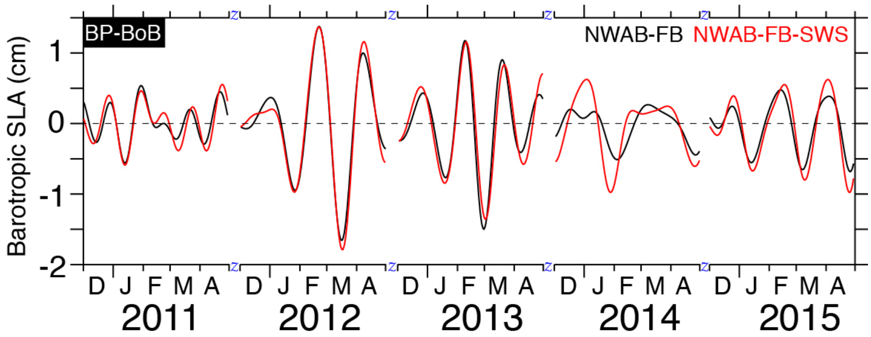

Supplementary Figure 6 | Flat bottom sensitivity experiments. Time series of intraseasonal barotropic SLA obtained from NWAB-FB experiment (black) compared with NWAB-FB-SWS experiment (red) at BP-BoB during the period December 2010 – April 2015. y=0 line is plotted for clarity. Supplementary Table 1 contains the details of the experiments. D, J, F, M, A in the x-axis labels represents December, January, February, March and April respectively.

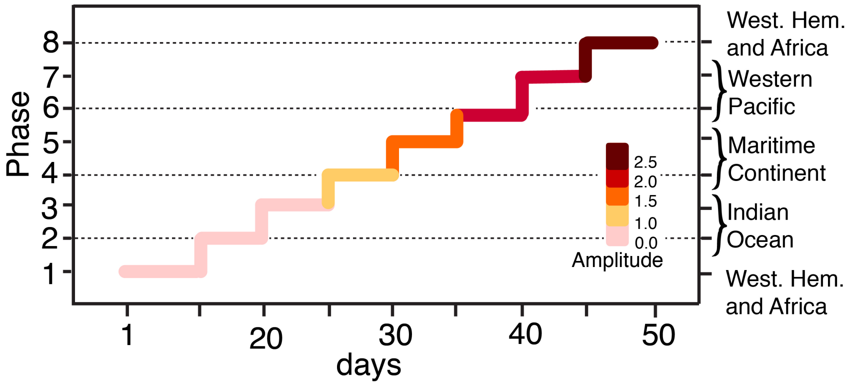

Supplementary Figure 7 | Schematic MJO phase diagram. Amplitude-phase schematic diagram for a sample MJO cycle having a periodicity of 50 days. Colors indicate the amplitude. Left y-axis represents the phases 1-8 in the MJO cycle. Right y-axis represents the typical location of the MJO in the Indo-Pacific basin during each of its phases.

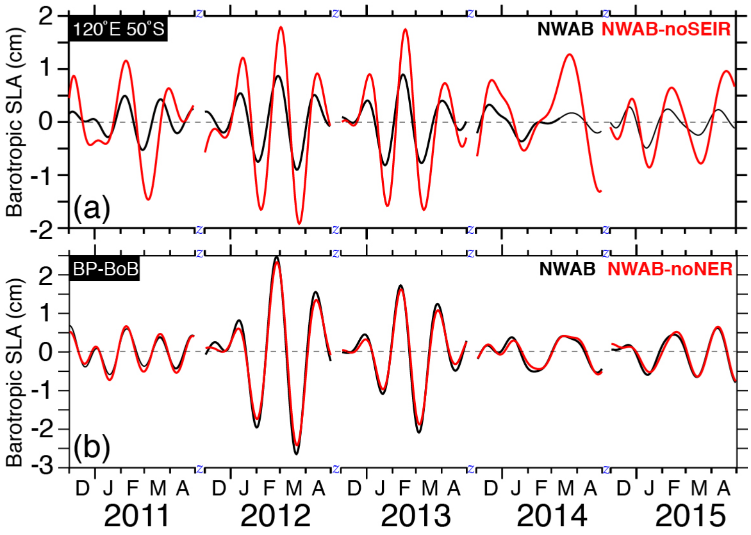

Supplementary Figure 8 | Influence of ridges. (a) Time series of intraseasonal barotropic SLA, obtained from NWAB (black) experiment, NWAB-noSEIR (red) experiment at 120° E,50° S during the period December 2010 – April 2015. (b) Time series of intraseasonal barotropic SLA obtained from NWAB(black) experiment and NWAB-noNER (red) experiment at BP-BoB during the period December 2010 – April 2015. y=0 line is plotted for clarity. Supplementary Table 1 contains the details of the experiments. D, J, F, M, A in the x-axis labels represents December, January, February, March and April respectively in panels (a) and (b).

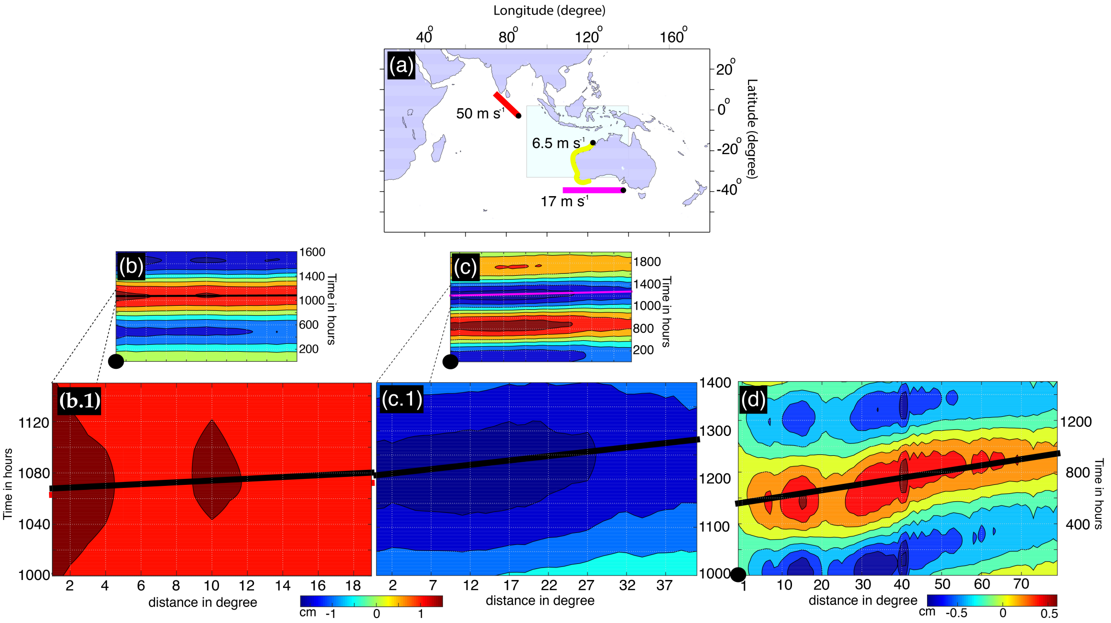

Supplementary Figure 9 | Inference of phase speeds of the barotropic waves. Hovmöller diagrams of model barotropic SLA extracted along the propagatory path of the waves (**a**) The three thick lines represent the sections used to compute the phase speeds of the three kinds of wave discussed in the paper. The red section is for the planetary Rossby wave, the pink section is for the topographic Rossby wave and the yellow section is for the continental shelf wave. The numbers represent the speed for each of the waves computed using Radon transformation. The shaded patch represents the forcing region in the NWAB experiment. The Hovmöller diagrams for (**b**) planetary Rossby wave, (**b.1**) close up view of (**b**), (**c**) topographic Rossby wave, (**c.1**) close up view of (**c**), and (**d**) continental shelf wave. The slope of the line in each of the Hovmöller diagrams determines the phase speed of the wave.

Name	Forcing Region	Stratification	Topography	Rotation	Purpose
**NWAB**	NWAB	Realistic	Realistic	varying *f*	To validate if NWAB is the prominent forcing region for large-scale coherent barotropic SLA variability in TIO.
**NWAB-NS**	NWAB	Non-stratified (T and S uniformly imposed to respective global average of 15°C and 35 psu)	Realistic	varying *f*	Observe effects of stratification on barotropic SLA and rule out the existence of baroclinic waves.
**NWAB-FB**	NWAB	Realistic	Three experiments with flat bottom (1100 m, 3000 m and 5300 m)	varying *f*	Observe effect of topographic gradients and depth on barotropic SLA and diagnose the physical nature of the various waves.
**NWAB-NR**	NWAB	Realistic	Real bathymetry at NWAB but flat bottom (5300 m) outside NWAB	varying *f*	Observe the role of topography outside NWAB in stearing the TRW and PRW.
**NWAB-CF**	NWAB	Realistic	Realistic	*f*-plane (-3.9 x 10^{-5} s^{-1})	Diagnose the physical nature of the various waves.
**NWAB-FB-SWS**	NWAB wind stress scaled by $H_0/H(x,y)$	Realistic	Flat bottom (H_0 = 3000 m)	varying *f*	Influence of topography on baroclinic-barotropic conversion.
**NWAB-noSEIR**	NWAB	Realistic	Realistic, except South East Indian Ridge (SEIR) removed	varying *f*	Diagnose the role of SEIR.
**NWAB-noNER**	NWAB	Realistic	Realistic, except Ninety east Ridge (NER) removed	varying *f*	Diagnose the role of NER.

Supplementary Table 1 | Model Sensitivity experiments. This table describes all the sensitivity experiments carried out using MOM4p1. The details of each experiment is outlined along the rows of the table corresponding to each such sensitivity experiment including the purpose to conduct such an experiment.
