# Supplementary material for: Basin-wide sea level coherency in the tropical Indian Ocean driven by Madden–Julian Oscillation
Source: Nat Commun. 2019 Mar 19;10:1257. doi: 10.1038/s41467-019-09243-5 (PMC6425029; doi:10.1038/s41467-019-09243-5)
Supplement: Supplementary file 2 — Description of Additional Supplementary Files [file 41467_2019_9243_MOESM2_ESM.docx]

**Description of Additional Supplementary Files**

File Name: Supplementary Movie 1

Description: **Wave propagation in the NWAB experiment during January-April, 2012:** (a) Time series of barotropic SLA at the BP-BoB location from NWAB experiment. The red and blue dashes emphasize the positive and negative phases of barotropic SLA. Time is reflected by the moving star on the curve. (b) Evolution of barotropic SLA from the NWAB experiment. Three *f/H* contours are also depicted in green(dotted), blue(dotted) and black(solid) lines. The black rectangular box represents the forcing region. The black star represents the BP-BoB location. (c) Evolution of intra-seasonal filtered $\left( \nabla\times\frac{\boldsymbol{\tau}}{H} \right)$ .

File Name: Supplementary Movie 2

Description: **Wave propagation in the NWAB-CF (*f* at 15^o^ S) experiment during January-April, 2012:** (a) Time series of barotropic SLA at the BP-BoB location from NWAB-CF experiment. The red and blue dashes emphasize the positive and negative phases of barotropic SLA. Time is reflected by the moving star on the curve. (b) Evolution of barotropic SLA from the NWAB-CF experiment. Blue dotted curves and black solid curves represent *f/H* contours. The black rectangular box represents the forcing region. The black star represents the BP-BoB location. (c) Evolution of intra-seasonal filtered $\left( \nabla\times\frac{\boldsymbol{\tau}}{H} \right)$ .

File Name: Supplementary Movie 3

Description: **Wave propagation in the NWAB-FB (*H* = 3000 m) experiment during January-April, 2012:** (a) Time series of barotropic SLA at the BP-BoB location from NWAB-FB experiment. The red and blue dashes emphasize the positive and negative phases of barotropic SLA. Time is reflected by the moving star on the curve. (b) Evolution of barotropic SLA from the NWAB-FB experiment. Horizontal blue dotted line and black solid line represent *f/H* contours. The black rectangular box represents the forcing region. The black star represents the BP-BoB location. (c) Evolution of intra-seasonal filtered $\left( \nabla\times\frac{\boldsymbol{\tau}}{H} \right)$ .

File Name: Supplementary Movie 4

Description: **Wave propagation in the NWAB-NR experiment during January-April, 2012:** (a) Time series of barotropic SLA at the BP-BoB location from NWAB-NR experiment. The red and blue dashes emphasize the positive and negative phases of barotropic SLA. Time is reflected by the moving star on the curve. (b) Evolution of barotropic SLA from the NWAB-NR experiment. Blue dotted line and black solid line represent *f/H* contours. The black rectangular box represents the forcing region. The black star represents the BP-BoB location. (c) Evolution of intra-seasonal filtered $f\left( \nabla\times\frac{\boldsymbol{\tau}}{H} \right)$.
